# Unusual Presentations of Cancer in the Male Breast

**DOI:** 10.7759/cureus.80052

**Published:** 2025-03-04

**Authors:** Swati Sharma, Sai Swarupa Vulasala, Sherif Elsherif, Smita Sharma

**Affiliations:** 1 Radiology, University of Florida College of Medicine, Jacksonville, USA

**Keywords:** breast, breast imaging, cancer, male, radiology

## Abstract

Breast cancer is a frequently diagnosed cancer in women, but the incidence of male breast cancer has also shown an increasing trend. Male and female breast cancers have some similarities, including risk factors and breast symptoms. Invasive ductal carcinoma is the most common type of breast cancer in both males and females. Even though fewer men than women are affected by breast cancer, male breast cancers are diagnosed at a more advanced stage and frequently have worse prognosis than their female counterparts. Through this case series, we are sharing a few unusual presentations of cancers in the male breast. In this case series of three patients, we will discuss the similarities and differences in male and female breast cancer. We aim to highlight the need to identify men at high risk and the importance of investigating any breast symptoms in men promptly.

## Introduction

Breast cancer stands as one of the leading cancers worldwide, with an estimated 300,590 new cases in 2023 [1]. It primarily affects the female population, with men constituting 1% of breast cancers [2]. Literature in male breast cancer is limited, with no prospective randomized trials available. There has been tremendous research and advancements in breast cancer diagnosis and treatment, leading to significantly improved prognosis and survival rates among women; however, trends in men have lagged behind [3]. It is, therefore, critical to carefully address any breast mass or breast-related symptoms in men without delay [4].

There are some common risk factors in males and females. Risk factors for male breast cancer (MBC) include age over 50, Black ethnicity, Ashkenazi Jewish ancestry, obesity, and genetic predispositions such as BRCA1/BRCA2 mutations and Klinefelter syndrome. Additional risk factors include a family history of breast or ovarian cancer, hormonal imbalances from estrogen therapy or liver disease, and prior chest radiation exposure [2,5].

Gynecomastia is seen in association with MBC; however, it is also very common in those without cancer and has not been established as a risk factor [6]. There are many histopathological types of MBC reported in the literature, including but not limited to invasive ductal carcinoma, ductal carcinoma in-situ, papillary carcinoma, invasive lobular carcinoma, lymphoma, and metastases from extramammary tumor [7]. Like their female counterparts, invasive ductal carcinoma (IDC) is the most common histological type among MBCs, observed in 80% of cases [5]. Around 35-50% of IDC demonstrate an associated ductal carcinoma in situ (DCIS) component on histopathological analysis [8]. The lobular histology is much rarer (0.5-1.5%) in men, as lobules do not typically form in male breasts [7].

Primary breast lymphoma (PBL) is a rare histological type, constituting 0.5% of breast cancers [9]. PBL is an infiltrating breast lymphoma with or without regional lymph node involvement in a patient with no history of prior nodal or extra-nodal lymphoma and lacking systemic disease at the time of diagnosis [10]. Among PBL, diffuse large B-cell lymphoma (DLBCL) is the common subtype, followed by follicular and marginal zone lymphoma [9]. So far, approximately 32 cases of male breast Non-Hodgkin’s Lymphoma (NHL) and only 14 cases of DLBCL have been reported in the literature [9,11].

Bilateral synchronous breast cancer is a rare presentation, accounting for 1%-3% of all breast cancers [12]. Bilateral synchronous breast cancer is defined as the presence of primary breast cancer in both breasts, diagnosed around the same time or within six months of each other. Bilateral synchronous breast cancer in a male is a unique condition, with limited case reports and without a documented incidence rate due to its rarity.

Paget’s disease of the breast, which is the involvement of the skin of the nipple and areola by adenocarcinoma, is a rare entity, usually associated with underlying DCIS and/or IDC. It represents 1%-3% of female breast cancers [13]. Paget’s disease of the male breast is quite rare, with a few cases reported in the literature.

Our report is a case series of three unusual biopsy-proven cancers in the male breast; the first is a case of PBL, the second is a case of bilateral breast cancer, and the third is a case of Paget’s disease of the breast with underlying IDC.

## Case presentation

Case 1

A 61-year-old male with hypertension and type 2 diabetes mellitus presented to his primary care physician with a one-week history of a non-tender palpable left breast mass. He denied fever, trauma, lymph node enlargement, night sweats, weight loss, and loss of appetite. On physical examination, there was a 2 cm mass in the outer left breast, without evidence of nipple retraction or skin thickening.

**Figure 1:**
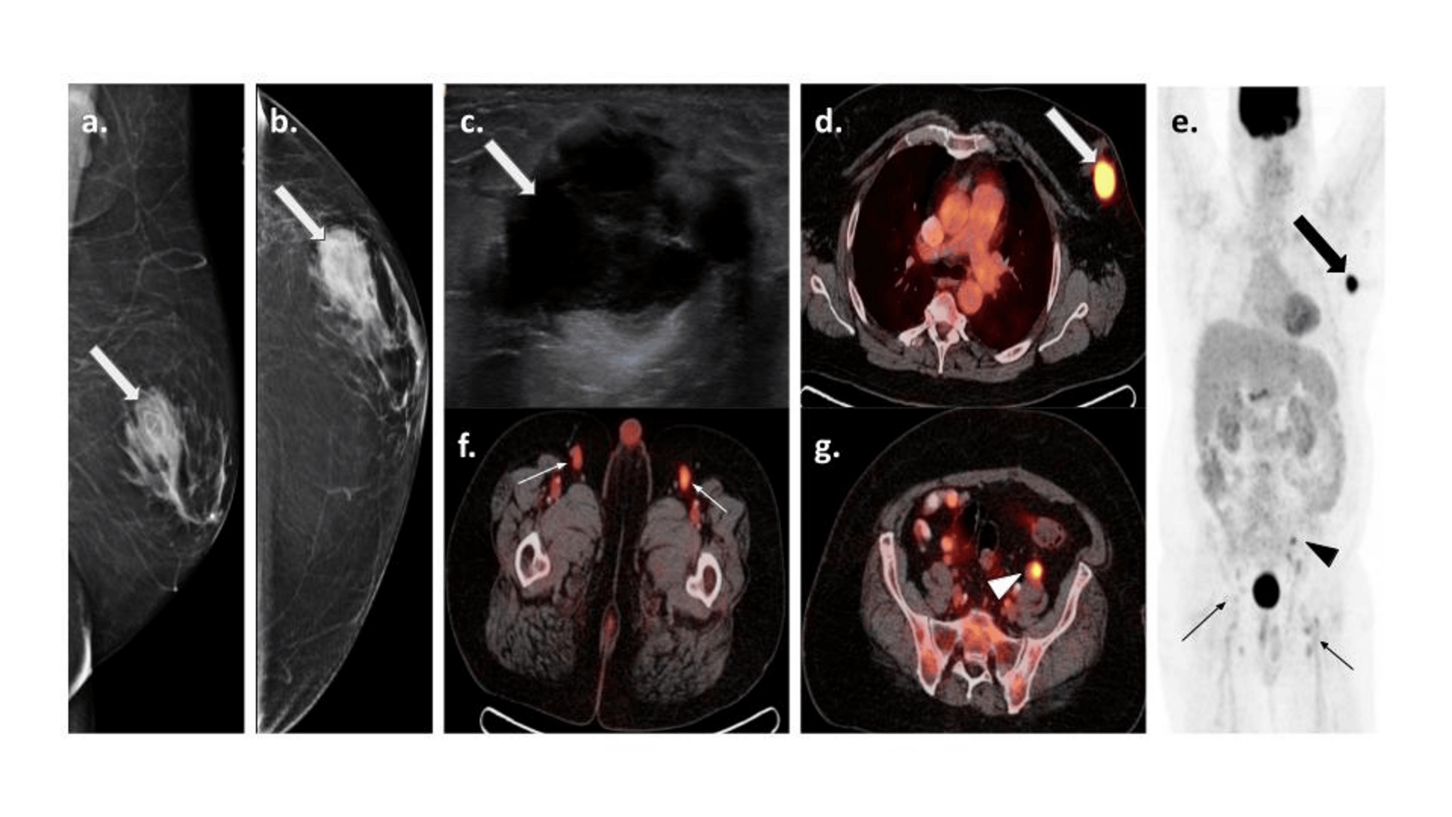
A 61-year-old male presented with a palpable left breast mass. Mediolateral oblique (MLO) (a) and cranio-caudal (CC) (b) views of mammogram show a mass (white arrows) in the outer central left breast at 3:00 o’clock, 2 cm from the nipple. Targeted grayscale ultrasound image of the left breast (c) shows a complex solid and cystic mass (white arrow) with thick internal septations. Ultrasound-guided biopsy yielded DLBCL. (d-h) PET/CT images show the metabolically active (SUV max 17.86) left breast mass (white arrow on (d) and black arrow on (e)), correlating to the biopsied lymphoma. Additionally, there are multiple metabolically active left common iliac (SUV max 4.57) (black arrowhead on (e) and white arrowhead on (g)) and bilateral inguinal (SUV max 4.73) (thin black arrows on (e) and thin white arrows on (f)) lymph nodes.

A diagnostic mammogram (Figures 1a, 1b) revealed bilateral gynecomastia and a 3.5 cm left breast mass at the 3:00 o’clock position, 3 cm from the nipple in the area of palpable concern. There was no associated nipple retraction, skin thickening, or axillary adenopathy. A targeted left breast ultrasound (Figure 1c) demonstrated a 3 x 1.9 x 3.3 cm complex cystic solid mass. There were no suspicious left axillary lymph nodes. Ultrasound-guided biopsy of the mass was performed, and it showed diffuse large B cell lymphoma (DLBCL) of the activated B-cell type. A subsequent bone marrow biopsy yielded multilineage hematopoiesis without lymphoid aggregates, acute leukemia, high-grade myelodysplasia, or overt immunophenotypic abnormalities.

Positron emission tomography/computed tomography (PET/CT) (Figures 1d-1g) was performed for staging, showing the biopsy-proven left breast lymphoma with a maximum standard uptake volume (SUVmax) of 18 and multiple nodal groups involvement below the diaphragm, including bilateral inguinal, external iliac, and left common iliac region. Subsequently, the patient completed one cycle of dose-adjusted-etoposide + prednisone + vincristine + cyclophosphamide + doxorubicin + rituximab (DA-R-EPOCH), and six cycles of rituximab + cyclophosphamide + doxorubicin hydrochloride + vincristine sulfate + prednisone (R-CHOP) chemotherapy regimen. Follow-up PET/CT after two cycles of R-CHOP regimen demonstrated a significant decrease in the size and fluorodeoxyglucose (FDG) uptake of the breast mass and multi-station lymph nodes. After completion of chemotherapy, the PET/CT showed no active disease. Thereafter, the patient has been under active surveillance to monitor for signs of lymphoma. 

Case 2

A 74-year-old African American male with type 2 diabetes mellitus, coronary artery disease, congestive heart failure, hypertension, chronic kidney disease, and morbid obesity (BMI of 55.5) presented to his primary care physician with a two-month history of palpable left breast mass. There was no family history of cancer. Physical exam showed a non-tender mass in the upper outer left breast, 3-4 cm from the nipple.

**Figure 2:**
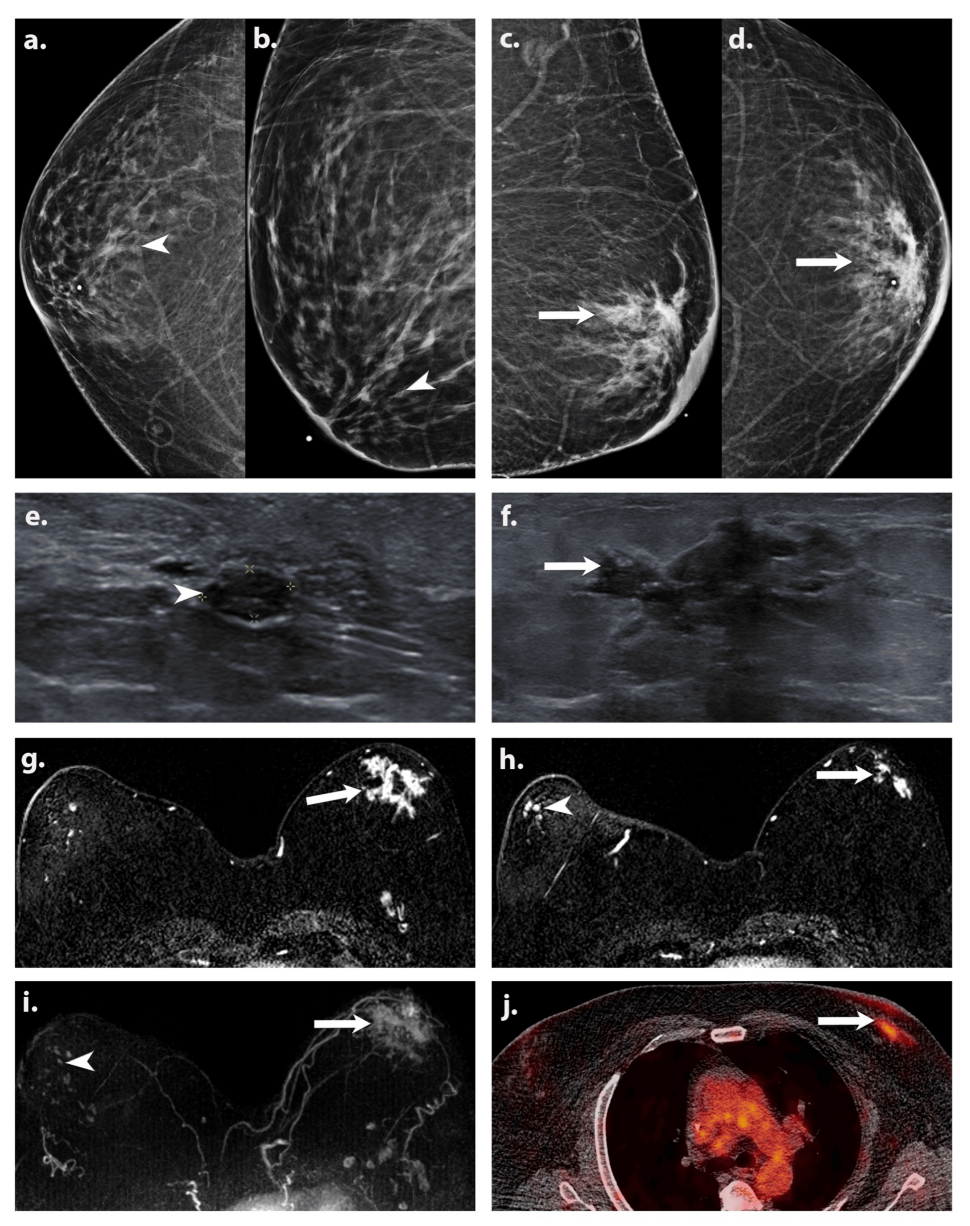
A 74-year-old African American male presented with a palpable left breast mass. Cranio-caudal (CC) and mediolateral oblique (MLO) views of a diagnostic mammogram (a-d) show suspicious microcalcifications within the retroareolar right breast and an irregular spiculated mass within the anterior left breast (white arrows) with overlying skin thickening. Targeted greyscale ultrasound (e) of the right retroareolar breast shows a solid hypoechoic mass (white arrowhead) at 11:00 o’clock, 1 cm from the nipple. Targeted greyscale ultrasound (f) image of the left retroareolar breast demonstrates an irregular spiculated hypoechoic mass (white arrow) from 11:00 to 1:00 o’clock, 3 cm from the nipple. Bilateral ultrasound-guided biopsy revealed left invasive ductal carcinoma (IDC), with associated high-grade ductal carcinoma in situ (DCIS) and right low-grade DCIS. Axial postcontrast T1-weighted fat sat (g,h) and MIP (i) images of the MRI show clumped non-mass enhancement within the left retroareolar breast (white arrows) with associated skin thickening. Segmental non-mass enhancement (white arrowhead) was noted in the right retroareolar region. Axial fused PET/CT image (j) shows hypermetabolic (SUVmax 4.53) left breast mass (white arrow) with skin thickening and minimal tracer uptake in the right breast tissue.

A diagnostic mammogram (Figures 2a-2d) revealed bilateral gynecomastia and an irregular spiculated mass with suspicious calcifications within the upper outer anterior left breast, with overlying skin thickening. A targeted ultrasound of the right breast (Figure 2e) revealed a 0.6 x 0.3 x 0.6 cm solid mass at 11 o’clock position, 1 cm from the nipple, which was subsequently biopsied, yielding ER-positive DCIS. Targeted ultrasound (Figure 2f) demonstrated an irregular spiculated hypoechoic left breast mass from 11:00 to 1:00 o’clock position, 3 cm from the nipple, and no suspicious left axillary lymph nodes. Ultrasound-guided biopsy was performed and showed moderately differentiated IDC with a component of DCIS. Immunohistochemistry showed positive estrogen receptor (ER), positive progesterone receptor (PR), positive Ki-67, and negative human epidermal growth factor receptor 2 (HER2). 

Subsequently, a Magnetic Resonance Imaging (MRI) of the breasts (Figures 2g-2i) was performed for evaluation of disease extent, and it demonstrated clumped non-mass enhancement within the left retroareolar breast with mixed kinetics, corresponding to the biopsy-proven cancer and skin thickening. The disease extent measured 7 x 7.1 x 6.2 cm. In addition, there was segmental non-mass enhancement with persistent kinetics within the right retroareolar region, 2.5 cm from the nipple, measuring up to 8.6 x 5.2 x 2.9 cm. This suspicious enhancement corresponded to right retroareolar calcifications, as detected on a retrospective review of a previously performed mammogram.

PET/CT (Figure 2j) showed bilateral hypermetabolic breast malignancy and no evidence of distant metastases. After the multidisciplinary management discussions among breast surgeons, oncologists, and radiation oncologists, the patient was started on neoadjuvant chemotherapy. The patient could only complete a single cycle of Docetaxel and cyclophosphamide due to intolerance. He was then started on tamoxifen and eventually proceeded to bilateral mastectomy with sentinel lymph node biopsy and axillary lymph node dissection. The final pathology of the resected right breast showed grade II DCIS with negative margins and no involvement of the two sentinel lymph nodes. The final pathology of the resected left breast revealed grade II IDC with negative margins and negative nodal involvement of the removed twelve axillary lymph nodes. The patient received post-mastectomy radiation therapy. Currently, the patient is under the care of hematology oncology and radiation oncology services.

Case 3

A 41-year-old African American male with morbid obesity (BMI of 55) presented to the breast surgeon with a one-year history of spontaneous clear to bloody left nipple discharge and left nipple rash. As per the patient, the rash did not respond to ointment containing neomycin, bacitracin, and polymyxin antibiotics. Family history was significant for breast and ovarian cancer in the paternal aunt and pancreatic cancer in the paternal grandfather. On physical examination, there was excoriation and rash on the left nipple and expressible serous to bloody nipple discharge. There was no palpable mass in the left breast. A punch biopsy of the left nipple performed by the breast surgeon revealed Paget’s disease with DCIS arising within a papillary lesion. Diagnostic breast imaging was ordered for further evaluation.

**Figure 3:**
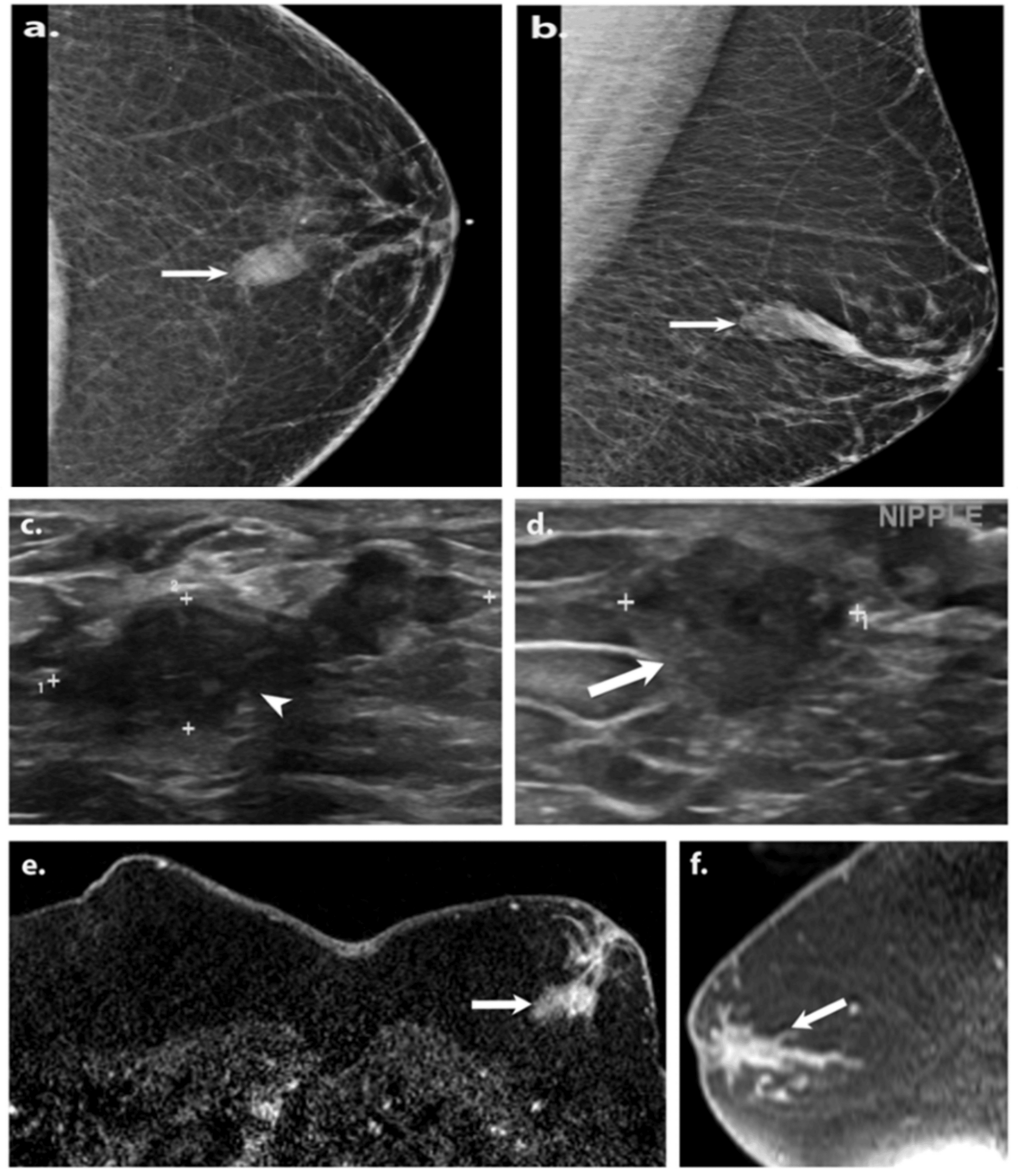
A 41-year-old African American male presented with intermittent left nipple discharge for one year. Cranio-caudal (CC) and mediolateral oblique (MLO) views of the diagnostic mammogram (a, b) show a tubular mass in the left retroareolar breast, likely representing a dilated duct (white arrows). Targeted greyscale ultrasound (c, d) images of the left retroareolar region at 11:00 o’clock demonstrates dilated duct (white arrowhead) with an irregular hypoechoic intraductal mass (white arrow). Left nipple punch biopsy revealed Paget disease of the breast and ductal carcinoma in situ, and subsequent ultrasound-guided biopsy showed invasive ductal carcinoma. Axial (e) and sagittal (f) postcontrast T1-weighted fat sat MRI images show an irregular heterogeneously enhancing mass in the left retroareolar breast (white arrows) involving the skin and nipple.

A diagnostic mammogram (Figures 3a, 3b) revealed a tubular mass in the left retroareolar breast suggestive of a dilated duct. Targeted left breast ultrasound (Figures 3c, 3d) of left retroareolar region at 11:00 o’clock position revealed irregular hypoechoic intraductal mass measuring 2.8 x 3.5 x 1 cm. There were no suspicious axillary lymph nodes. Ultrasound-guided biopsy was performed and revealed poorly differentiated IDC with a DCIS component. Immunohistochemistry showed positive ER, positive Ki-67, negative PR, and negative HER2.

MRI of the breasts (Figures 3e, 3f) showed an irregular heterogeneous enhancing mass in the left retroareolar breast with washout kinetics, involving the skin and nipple, and with a total extent of approximately 6.3 x 3.1 x 3.3 cm. Genetic testing was positive for Partner and Localizer of BRCA2 (PALB2) mutation. Patients with this rare genetic mutation are two to four times more likely to develop breast cancer. Thereafter, the patient underwent left therapeutic and right prophylactic mastectomy in addition to left sentinel lymph node biopsy. The final pathology of the left breast showed grade III invasive ductal carcinoma (IDC), ductal carcinoma in situ (DCIS), Paget disease of the breast, negative margins, and negative sentinel lymph nodes. Following surgery, he received radiation therapy to the left chest wall. Currently, he is under the care of radiation oncology and hematology oncology services. 

## Discussion

Despite its rarity, male breast cancer (MBC) has witnessed a 40% increase in incidence from 1975 to 2015, surpassing the 25% rise in females according to surveillance, epidemiology, and end results (SEER) data [14]. The American Cancer Society projects 2790 new MBC cases in 2023 [1]. Predominantly comprising invasive ductal carcinoma (IDC) at 80%, MBC also includes subtypes such as ductal carcinoma in situ (DCIS), invasive papillary carcinoma, Paget’s disease of the breast, and primary breast lymphoma [15,16].

Men diagnosed with breast cancer face unique challenges. The mean age for MBC diagnosis is five years older than in females, typically between the ages of 60 to 69, with the characteristic presentation of a painless palpable lump greater than 2 cm [17]. There may be associated palpable axillary lymph node, nipple discharge, nipple retraction or inversion, and skin thickening [18]. MBC is typically unilateral, with fewer than 1% of patients exhibiting bilateral disease [19]. When compared to females, MBC tends to present with larger tumor size, lymphadenopathy, and at a more advanced stage, largely attributed to the absence of screening and lack of general awareness due to low disease prevalence [18]. Mortality rates for cancers in male breasts have not significantly decreased, with a reported five-year overall survival of 74%, lower than the 83% observed in females [18,20].

**Figure 4:**
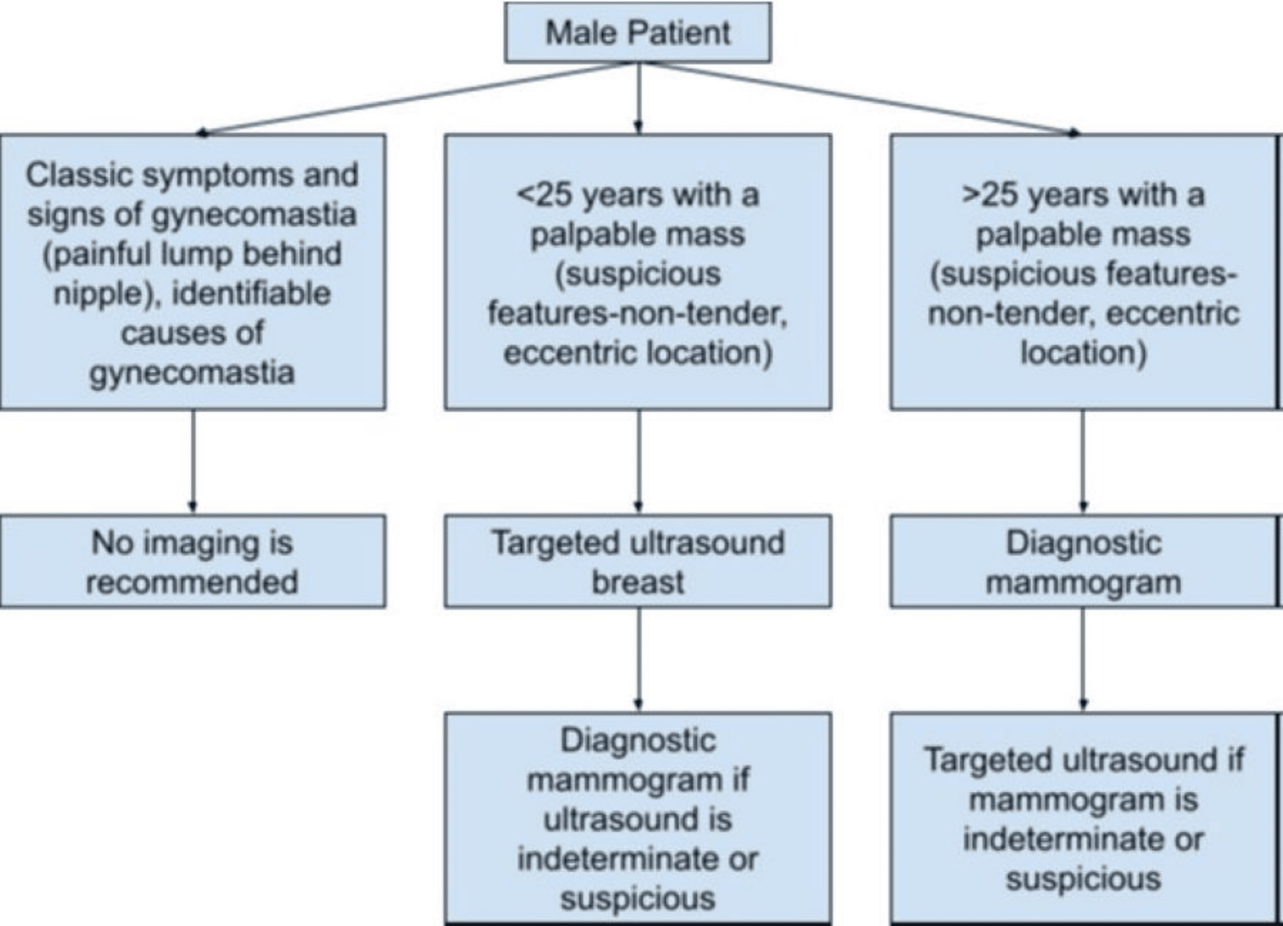
Workup of male breast symptoms. Imaging workup of male breast symptoms created by the authors utilizing American College of Radiology appropriateness criteria [21].

Symptomatic patients are evaluated with diagnostic mammography and targeted ultrasound (Figure 4), based on the American College of Radiology appropriateness criteria [21]. Diagnostic mammography is highly sensitive (92-100%) and specific (90-96%) in detecting breast abnormalities and has a 99-100% negative predictive value [18]. Mammographically, MBC may be seen as a mass with or without calcifications [22,23]. Gynecomastia, a common etiology of palpable lumps in male breasts, is commonly a painful condition (Figure 4). Gynecomastia can be bilateral, is often asymmetric, and is classically retroareolar and of equal density. Distinguishing features of MBC from benign gynecomastia include irregular or spiculated high-density mass, eccentric location, and secondary features such as nipple inversion and skin thickening [22]. Calcifications are infrequent in MBC. However, unlike females, where calcifications can frequently be attributed to benign entities, calcifications in male breasts are almost always suspicious. The exceptions to this rule are typically benign vascular or dermal calcifications [24]. Calcifications associated with MBC are usually microcalcifications and occur in the DCIS component of IDC [25]. Ultrasound appearance of MBC mirrors that in females, presenting as a solid, hypoechoic, irregular, non-parallel, and non-circumscribed mass located in the subareolar region [22]. Axillary ultrasound is crucial, as 47% of MBCs have axillary lymph node involvement at diagnosis [22]. MRI in MBC, though not essential before histologic diagnosis, is significant for evaluating disease extent and identifying occult lesions in the contralateral breast, as is the case in female breast cancers [21].

Treatment strategies for MBC include surgery, hormonal therapy, chemotherapy, and radiation, depending on the disease stage. There is a high prevalence of hormone-receptor-positive disease, emphasizing the pivotal role of hormone therapy in MBC treatment. Post-treatment, men with breast cancer have a 30-fold greater risk of contralateral breast cancer [26]. After definitive management, a consensus by the American Society of Clinical Oncology recommends an annual mammogram for men with predisposing mutation and a personal history of breast cancer [27].

Considering the escalating prevalence of MBC, discussions revolve around whether screening should be considered in males akin to females [17]. Organizations such as the American Cancer Society and the National Comprehensive Cancer Network advocate for clinical breast exams in high-risk men, but specific guidelines for image-based screening in asymptomatic individuals are lacking. Some studies highlight the potential of screening mammograms in detecting MBC at an earlier stage among high-risk individuals [18]. The drawbacks of screening mammograms are false positives in patients with gynecomastia, financial considerations in establishing and maintaining a screening program, and the minimal but existing exposure to ionizing radiation [18].

Larger studies are required to provide more data for validating mammography screening in males, particularly in those at high risk, as well as to explore potential risk factors for MBC. Identifying at-risk males is a crucial step towards potential screening interventions in vulnerable populations, with the goals of preventing late-stage diagnosis and improving prognosis. A personal history of breast cancer, a strong family history, or Ashkenazi Jewish ancestry are established risk factors for MBC. Obesity and hyper-estrogenic states have also been recognized as risk factors. Further research to understand the potential influence of the current obesity pandemic on the rising incidence of MBC is warranted. Notably, two patients discussed in our study were morbidly obese at the time of presentation and diagnosis. Highlighting racial disparities, breast cancer incidence and mortality rates are notably higher in African American men compared to other ethnicities. Both cases of primary breast cancer in our report involved African American men. Genetic mutations, including but not limited to BRCA1 and BRCA2, increase the likelihood of MBC, as exemplified in one patient in our report with a PALB2 mutation.

In summary, the proactive identification of males at an elevated risk for breast cancer holds intrinsic value in improving prognosis and decreasing mortality rates in male breast cancer. Healthcare providers can help identify high-risk men by conducting a thorough evaluation of personal and family history. Subsequently, a comprehensive approach involving tailored counseling, regular physical exams, ongoing surveillance, and genetic evaluations can be considered, pending the validation of a formal screening program in these high-risk males.

## Conclusions

Cancer in the male breast is an uncommon disease with limited studies. Due to its increasing incidence and due to the worse prognosis and survival rates in males compared to females, there is a need to focus more on this entity and for further research. By sharing the unusual presentations of cancer in the male breast and by delineating the similarities and differences in male and female breast cancers, our goal is to engage the readers with this entity and to promote the need for immediate attention to breast symptoms in men.
